# Study of the whole genome, methylome and transcriptome of *Cordyceps militaris*

**DOI:** 10.1038/s41598-018-38021-4

**Published:** 2019-01-29

**Authors:** Yujiao Chen, Yuqian Wu, Li Liu, Jianhua Feng, Tiancheng Zhang, Sheng Qin, Xingyu Zhao, Chaoxia Wang, Dongmei Li, Wei Han, Minghui Shao, Ping Zhao, Jianfeng Xue, Xiaomin Liu, Hongjie Li, Enwei Zhao, Wen Zhao, Xijie Guo, Yongfeng Jin, Yaming Cao, Liwang Cui, Zeqi Zhou, Qingyou Xia, Zihe Rao, Yaozhou Zhang

**Affiliations:** 1 Human Genome Research Center, Tianjin University, Tianjin, 300309 China; 2 Zheng-Yuan-Tang (Tianjin) Biotechnology Co. Ltd, Tianjin, 300457 China; 3 Tianjin Lakeside Powergene Science Development Co. Ltd, Tianjin, 300309 China; 4 Zhejiang Chinagene Biomedicine Co. Ltd, Jiaxing, 314400 China; 5 Guizhou Gui’an Academy of Precision Medicine Co. Ltd, Gui’an, 561113 China; 6 State Key Laboratory of Silkworm Genome Biology, Southwest University, Chongqing, 400715 China; 7 College of Life Sciences, Jiangsu University of Science and Technology, Zhenjiang, 212000 China; 8 College of Life Science, Zhejiang University, Hangzhou, 310058 China; 9 Department of Biochemistry and Molecular Biology, China Medical University, Shenyang, 110001 China; 10 Department of Entomology, Penn State University, PA, 16802 USA; 11 Dynamiker Biotechnology (Tianjin) Co., Ltd, Tianjin, 300467 China; 12 Tianjin International Joint Academy of Biomedicine, Tianjin, 300457 China

## Abstract

The complete genome of *Cordyceps militaris* was sequenced using single-molecule real-time (SMRT) sequencing technology at a coverage over 300×. The genome size was 32.57 Mb, and 14 contigs ranging from 0.35 to 4.58 Mb with an N50 of 2.86 Mb were assembled, including 4 contigs with telomeric sequences on both ends and an additional 8 contigs with telomeric sequences on either the 5′ or 3′ end. A methylome database of the genome was constructed using SMRT and m4C and m6A methylated nucleotides, and many unknown modification types were identified. The major m6A methylation motif is GA and GGAG, and the major m4C methylation motif is GC or CG/GC. In the *C*. *militaris* genome DNA, there were four types of methylated nucleotides that we confirmed using high-resolution LCMS-IT-TOF. Using PacBio Iso-Seq, a total of 31,133 complete cDNA sequences were obtained in the fruiting body. The conserved domains of the nontranscribed regions of the genome include TATA boxes, which are the initial regions of genome replication. There were 406 structural variants between the HN and CM01 strains, and there were 1,114 structural variants between the HN and ATCC strains.

## Introduction

*C*. *militaris* is as highly valued in Chinese traditional medicine as the ascomycete *Cordyceps sinensis* (syn. *Ophiocordyceps sinensis*), which possesses antitumor properties^1^. There are currently more than 680 documented species of the ascomycete genus Cordyceps. *C*. *militaris*, which is a pathogen of the lepidopteran insect pupae^2,3^, has been successfully cultivated and grown on grain or *Bombyx mori* pupae. *C*. *militaris* HN is an edible fungus that was approved as the first novel food of the Cordyceps species by the Ministry of Public Health of China in 2009^1^. In recent years, advanced techniques have demonstrated that the nutrients and bioactive compounds in the fruiting body of *C*. *militaris* are similar to those of the traditional Chinese invigorant, *O*. *sinensis*^4,5^. Therefore, analyses of the *C*. *militaris* genome, transcriptome and methylome are important for understanding the biology of this fungus.

Of the available sequencing platforms, SMRT technology has the unique advantage of significantly longer read lengths that produce high-quality genomes^6^. Further, SMRT Iso-Seq has the great advantage of not requiring sequence assembly, thus increasing the integrity of the assembled transcriptome and the reliability of transcriptome sequencing^7,8^.

Recently, using a Roche 454 GS FLX system, the *C*. *militaris* genome was assembled at a 147× coverage into 597 contigs and 33 scaffolds with a scaffold N50 of 4.6 Mb and a total genome size of 32.2 Mb; however, due to the limitations of the sequencing technology used, several gaps remain in the assembled genome^9^. Using SMRT sequencing and Optical Mapping, the fungal genome of *V*. *dahliae* was assembled at the chromosome level^10^. To date, the genomes of 12 fungal species have been assembled at the chromosome level using SMRT sequencing^11–13^. In addition, the genome of the ATCC strain of *C*. *militaris* with 7 contigs has been reported^14^.

DNA methylation is among the most common forms of DNA modification in prokaryotic and eukaryotic genomes. DNA methylation has various effects on fundamental biological processes, including the silencing of transposable elements (TEs) and the regulation of chromatin structure, gene expression, genetic recombination and sexual development^15–17^. Bisulfite sequencing (BS-Seq) and SMRT technology have been widely used in the sequencing of the genomes and methylomes of fungi^18–20^. Based on the CM01 genome database^9^, the methylome of *C*. *militaris* at a single-base resolution has been used to assess the DNA methylation patterns during sexual development using genomic BS-Seq^17^. The results showed that approximately 0.40% of cytosines are methylated, which is similar to the DNA methylation level during asexual development (0.39%). More recently, in a study using SMRT technology, up to 2.80% of all adenines were methylated in 16 early-diverging fungi and N6-methyldeoxyadenine (6 mA) was identified as a widespread epigenetic marker in early diverging fungi that is associated with transcriptionally active genes^21^.

In this study, the genome, transcriptome and methylome of the *C*. *militaris* HN strain were assembled and analyzed. The genomic nontranscribed region structures were identified. The methylation types of genomic DNA on all four nucleotides were detected using high-resolution LCMS-IT-TOF. These results provide a new approach to performing relevant genomic studies.

## Results

### Sequencing and assembly of the *C*. *militaris* genome

**Figure 1:**
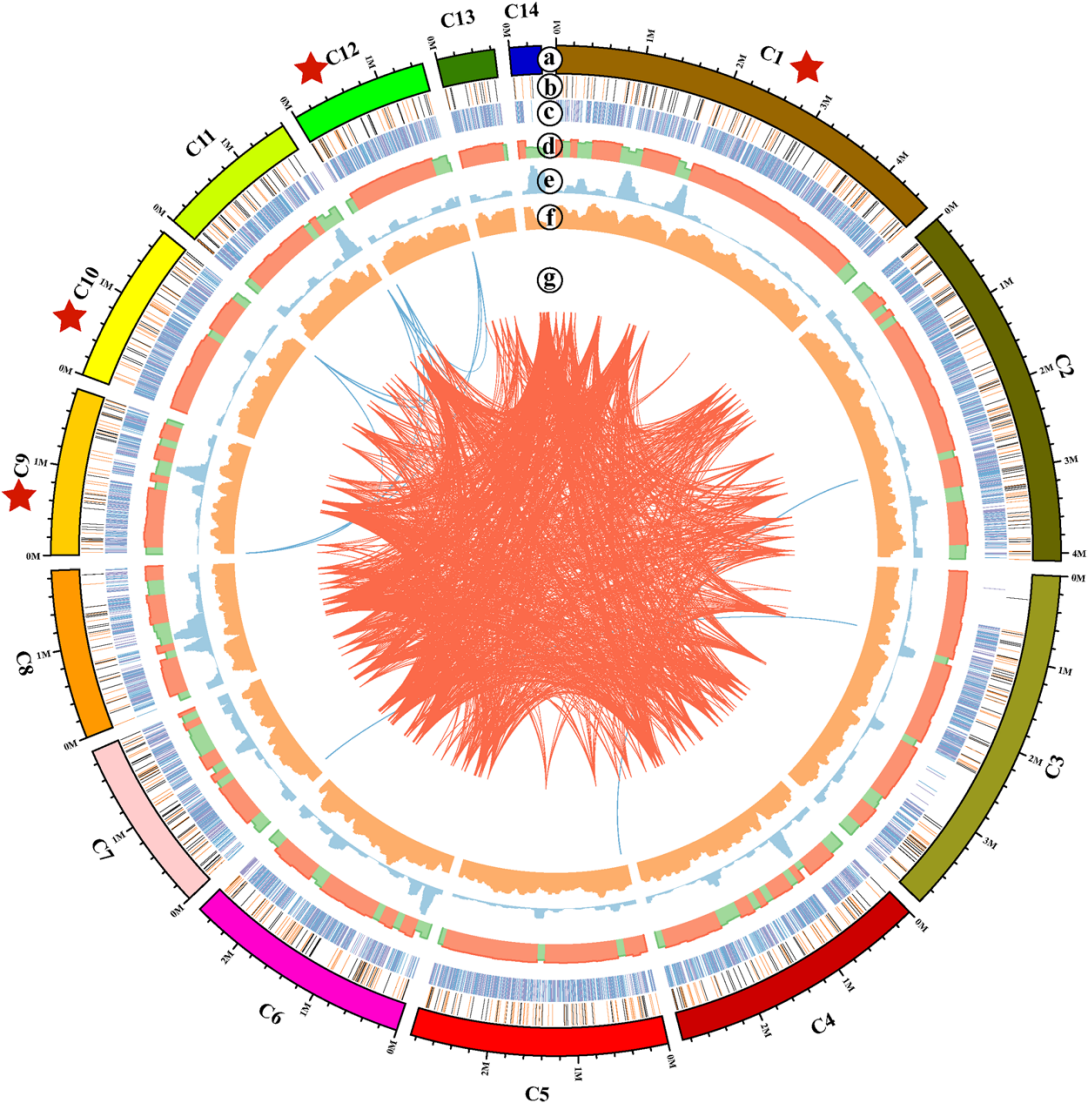
Characteristics of the *de novo* assembly genomic features in *C*. *militaris* (HN strain). (**a**) The 14 contigs with the complete chromosomes are labeled with red stars. (**b**) Plots of the m6A motif distributions. The orange color represents m6A in the plus strain, the brown color represents m6A in the minus strain and the black color shows the overlap of brown with orange and shows that m6A exists in both strains. (**c**) Plots of the m4C motif distributions. The purple color represents m4C in the plus strain and the blue color represents m4C in the minus strain. (**d**) The distribution of the GC content: the red color represents a GC content >50% and the green color represents a GC content <50%. (**e**) Density distribution of repeat elements. (**f**) Density distribution of genes. (**g**) Genome duplication: regions sharing >90% sequence similarity over 5 kb are connected by red lines; those with >90% similarity over 10 kb are connected by blue lines.

We assembled the genome using the Hierarchical Genome Assembly Process 3 (HGAP3) of SMRT^6^. More than 300× coverage of the *C*. *militaris* genome was achieved, with an average polymerase read length of 14 kb. The *C*. *militaris* genome was assembled into 14 contigs, and the total genome size was 32.57 Mb. The contig sizes ranged from 0.35 to 4.57 Mb, and the contig N50 was 2.86 Mb (Fig. 1, Table 1). Of the 14 contigs, contigs 1, 9, 10 and 12 contained GGGTAA or TTACCC telomeric repeat sequences of approximately 120 bp in length on both ends, indicating that the four contigs were complete chromosomes. Eight additional contigs contained telomeric repeat sequences on the 5′ or 3′ end (Fig. 1a). The distribution of DNA methylation is shown in Fig. 1b,c. The GC content in the *C*. *militaris* HN strain genome was 51.5% and was not evenly distributed among the individual contigs (Fig. 1d). Contig 14 was unique in terms of its GC content, and 2/3 of the contig had less than 40% GC content. Additionally, the frequency of repeat sequences was higher in regions with a lower GC content combined with a lower frequency of coding sequences (Fig. 1d–f). Such regions may function as gene regulatory regions or chromosomal regions with an ultra-complex structure. The *C*. *militaris* genome has many genome duplications greater than 5 kb (Fig. 1g).

**Table 1:** Assembly summary statistics of *C*. *militaris* HN compared with the ATCC 34164 and CM01 *C*. *militaris* genomes.

	*Cordyceps militaris* HN	*Cordyceps militaris* ATCC 34164	*Cordyceps militaris* CM01
Assembly size (MBa)	32.57	33.62	32.27
Sequencing platform	PacBio RS II	PacBio RS II	Roche 454
Coverage fold	317.7x	149.5×	147x
Number of Scaffolds (Contigs)	14	7	31/597
N50 (Mb) Scaffold/Contigs	2.86	5.78	4.55/0.11
GC Content (%)	51.55	50.92	51.41
Repeat Content (%)	7.72	9.41	8.10
Predicted Genes	10095	9287	9651
Number of Exons	29663	26026	28872
Number of Introns	19568	16739	19221
Total Gene Length(Mb)	18.9	16.1	16.8
Mean Intergenic Region Length	1432	1866	1596
Gene Density (Genes/Mbp)	309.9	278.9	301
Mean Gene Length	1872/1385	1739	1743
Mean Exon Length	471	550	507
Mean Intron Length	116	109	113
Mean Introns Per-gene	1.9	1.8	2.0
NCBI Accession	SUB1679810	PRJNA323705	PRJNA225510

We compared our genome database with the database of the CM01 strain of *C*. *militaris* from the Roche 454 GS FLX platform^9^, and the number of contigs in the genome was reduced from 594 to 14. N50 and the genomic size increased 26-fold and by 0.3 Mb. As shown in Table 1, the average gene length increased by 128 bp, the protein coding genes increased by 411 bp and the average intergenic length decreased by 226 bp. We also compared our genome with the recently released genome of an ATCC strain sequenced by PacBio sequencing technology; as shown in Table 1, the genomic size decreased by 1.05 Mb, the number of genes increased by 808, the average intergenic length decreased by 434 bp, and the number of exons increased by 3,637.

**Figure 2:**
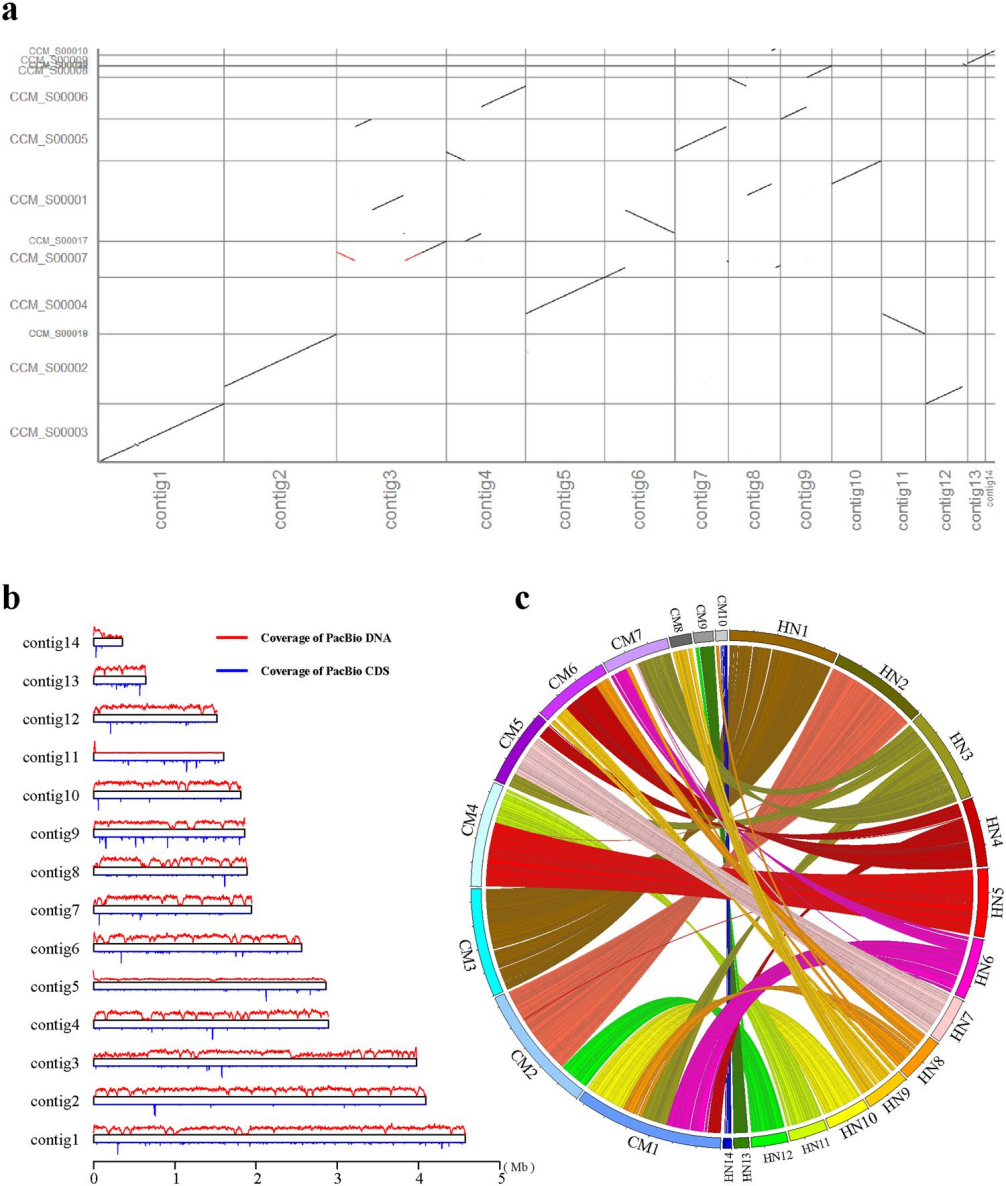
Comparison of the SMRT assembly to a previous CM01 genome and sequencing depth distribution of the genome and transcriptome. (**a**) Dot plots comparing the SMRT assembly to a previous CM01 genome identified large genomic variants between the two strains. (**b**) The distribution of the sequencing depth of the genome and transcriptome. (**c**) Contigs in HN mapped to the previous CM01 genome.

SMRT sequencing of the *C*. *militaris* genome revealed many interchromosome translocations from the shotgun CM01 sequencing database (Fig. 2a,c). As shown in Fig. 2a,c, contig 3 of the HN strain genome is composed of scaffolds 1, 5 and 7 from the CM01 genome; contig 4 is composed of scaffolds 1, 5 and 6; contig 7 is composed of scaffolds 1 and 7; and contig 8 is composed of scaffolds 1, 6, 7 and 10. Contig 4 is a part of scaffold 4 with an inverse direction. The coverage distribution of the genome and transcriptome sequencing were also investigated. Compared with the other contigs, contigs 5 and 11 had lower coverage, suggesting that these two contigs have distinct spatial structural features. Furthermore, this finding suggests that the gaps in the genome are not due exclusively to random repeat sequences or a high GC content, and many unknown factors must be considered (Fig. 2b). The translocations between the genome of the HN and ATCC strains were also investigated, and we found that contig 3 of the HN genome existed as an inverted duplication (Supplement 1).

### DNA methylation analysis in the genome of the *C*. *militaris* HN strain

**Figure 3:**
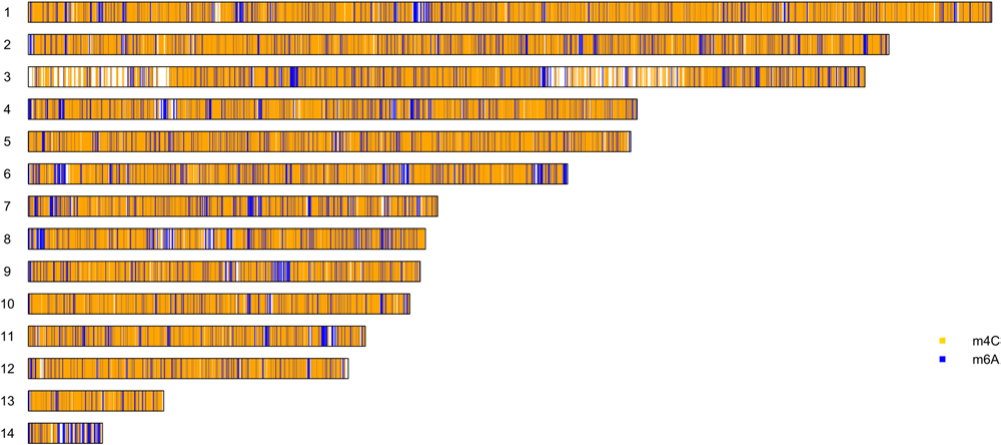
Distribution of m4C and m6A methylated sites in 14 contigs in the HN genome.

**Figure 4:**
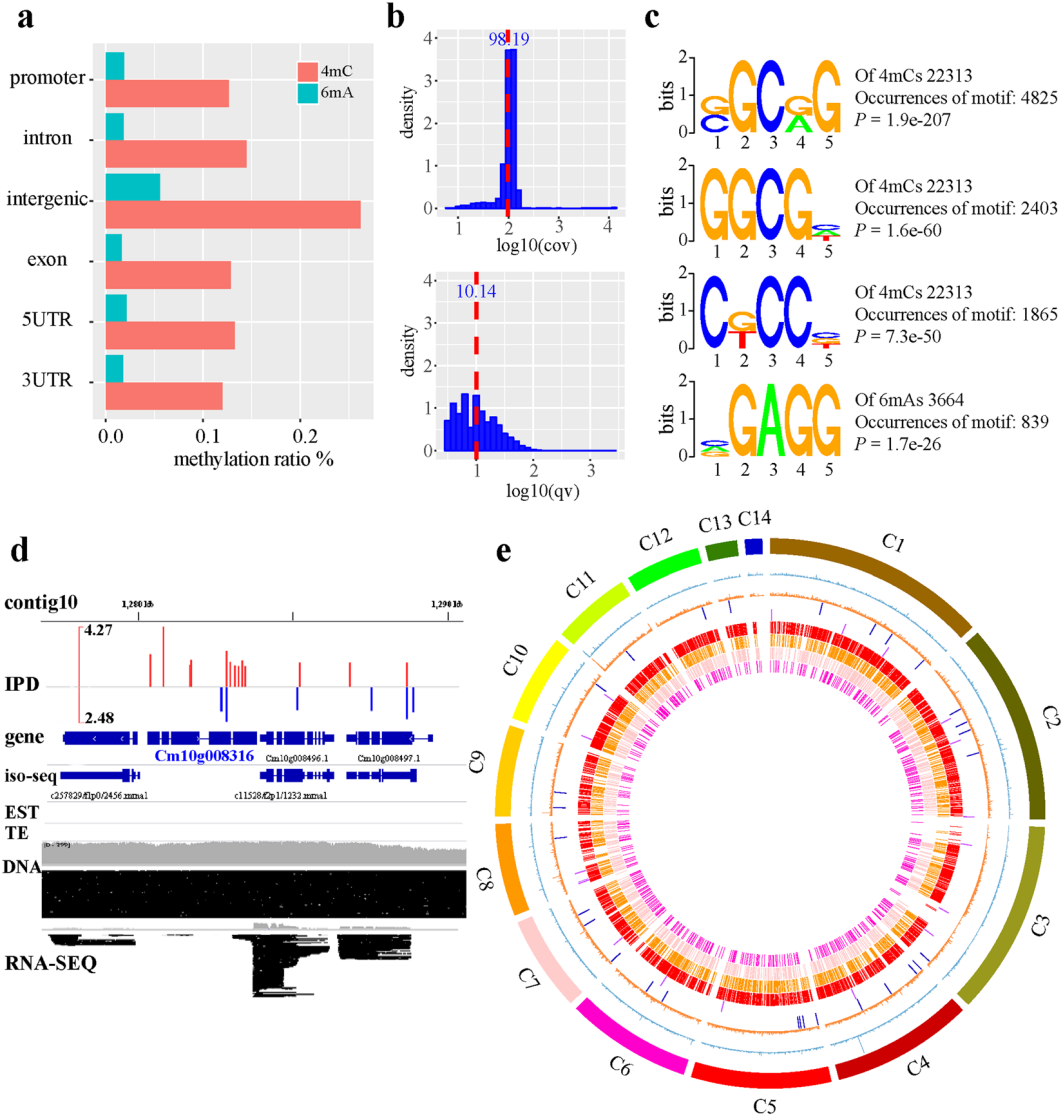
Distribution of methylation in the HN genome. (**a**) The distributions of m4C and m6A in different parts of the genome. (**b**) Density distribution of coverage and quality in the m4C and m6A motifs. (**c**) SMRT sequencing identified motifs associated with m6A or m4C. NGCNC, GGCG and CNCCN are associated with m4C methylation. NGAGG is associated with m6A methylation. (**d**) Representative interpulse duration (IPD) ratios of SMRT sequencing data of the gene Cm10g008316. IPD ratio is defined as the change in the IPD distribution in the sample compared with the unmodified bases. Red, positive strand; blue, negative strand. (**e**) Circos plots of m4C, m6A and motif distributions; from outer ring to inner rings: the density distribution of m6A, the density distribution of m4C, the genome location of cordycepin pathway genes, the location of the ergosterol pathway genes in the genome, genomic location of the NGCNG motif in m4C, genomic location of the GGCGN motif in m4C, genomic location of the CNCCN motif in m4C, and genomic location of the NGAGG motif in m6A.

**Figure 5:**
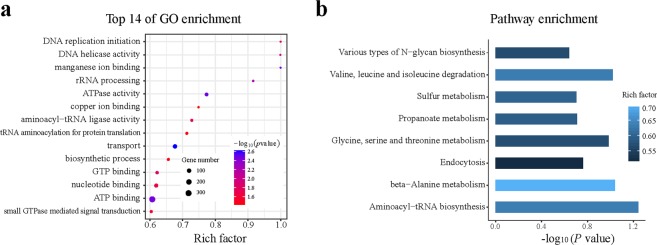
Annotation of the methylated genes in the HN genome. (**a**) GO annotation information of the methylated genes and top 14 GO enrichment terms. (**b**) KEGG annotation information of the methylated genes.

The methylome and its distribution on the 14 contigs of the *C*. *militaris* genome were also determined by SMRT sequencing (Fig. 3). Two major types of methylation, including m4C and m6A, were identified, and their distribution patterns are shown in Fig. 4. The distributions of the methylated nucleotides among the different contigs are shown in Table 2. In total, 0.016% and 0.085% of m6A and m4C were observed in contigs 1 and 13, respectively, while contig 14 contained 0.032% of m6A and 0.042% of 4mC. An in-depth analysis of the m6A methylation motifs in the contig showed that GA is the most common motif, accounting for 80% of all methylation sites, including GAG, GGA and GGAG at 6%, 23% and 17%, respectively (Fig. 4c). The GO and KEGG annotation information for the methylated genes and the top 14 GO enrichment terms are shown in Fig. 5.

**Table 2:** Distributions and methylation motifs in 14 contigs in HN.

Contigs	Total length	Number of m6A (%)	Number of m4C (%)	Number of unknown (T or G) (%)
contig1	4576244	682 (0.0149)	4067 (0.0889)	98395 (2.1501)
contig2	4088212	694 (0.017)	3658 (0.0895)	87643 (2.1438)
contig3	3973962	524 (0.0132)	2503 (0.063)	57715 (1.4523)
contig4	2891972	508 (0.0176)	2491 (0.0861)	56909 (1.9678)
contig5	2861787	433 (0.0151)	2778 (0.0971)	65612 (2.2927)
contig6	2561359	464 (0.0181)	2177 (0.085)	50147 (1.9578)
contig7	1944127	392 (0.0202)	1580 (0.0813)	38851 (1.9984)
contig8	1886322	329 (0.0174)	1380 (0.0732)	34734 (1.8414)
contig9	1861270	311 (0.0167)	1593 (0.0856)	39143 (2.103)
contig10	1812096	278 (0.0153)	1746 (0.0964)	41142 (2.2704)
contig11	1600700	321 (0.0201)	1475 (0.0921)	32845 (2.0519)
contig12	1519743	197 (0.013)	1361 (0.0896)	32135 (2.1145)
contig13	643026	93 (0.0145)	611 (0.095)	13736 (2.1362)
contig14	352336	114 (0.0324)	147 (0.0417)	3021 (0.8574)
Total	32573156	5340 (0.0164)	27567 (0.0846)	652028 (2.0017)

### Genomic DNA methylation detected by LCMS-IT-TOF

**Figure 6:**
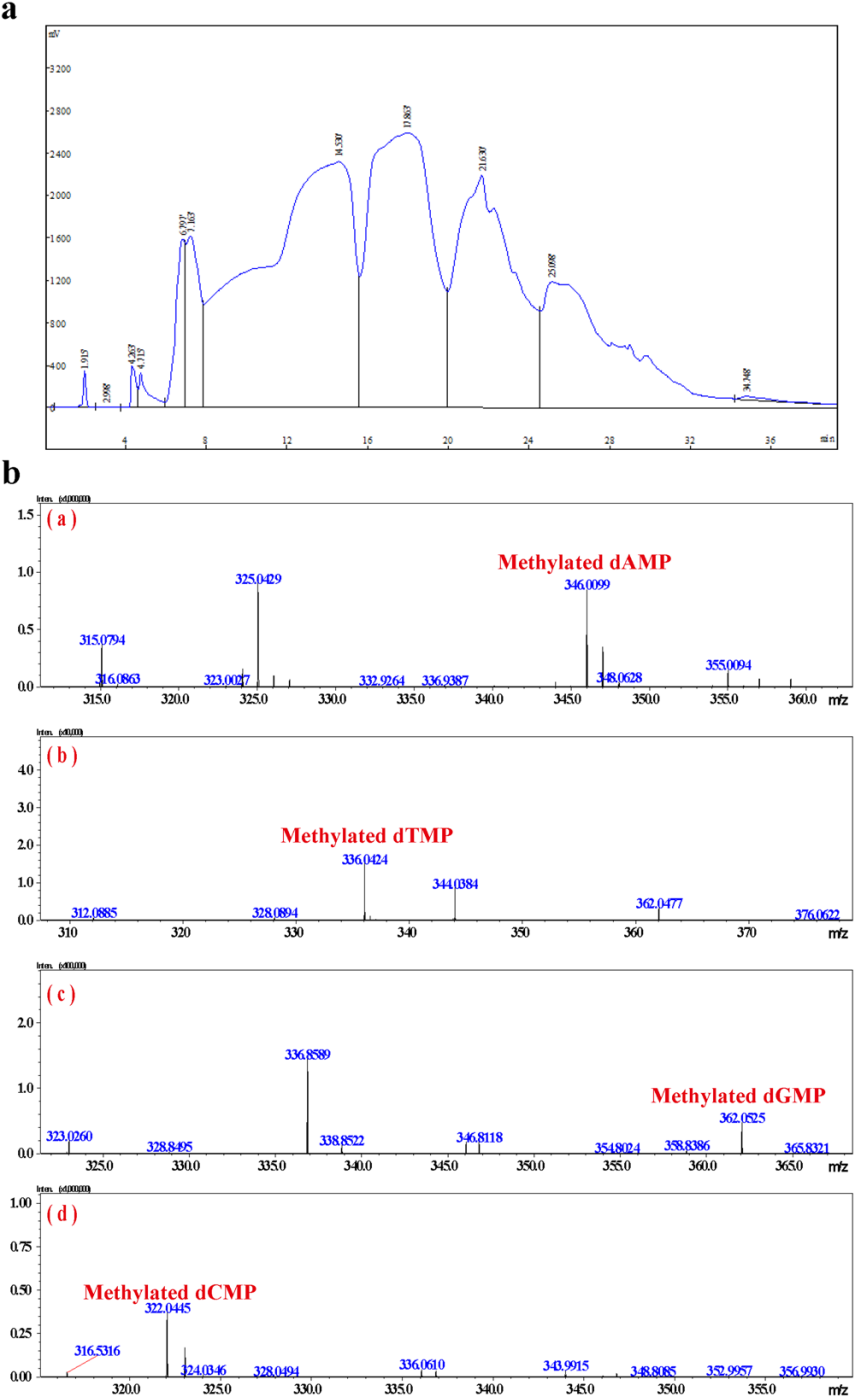
(**a**) Four types of nucleotides and hypothetical methylated nucleotides in the genomic DNA separated by HPLC. (**b**) Methylated single nucleotides in the genomic DNA as detected by MS based on their molecular weights.

To determine whether all 4 nucleotides were methylated in the *C*. *militaris* genome, the molecular weight of each nucleotide in the *C*. *militaris* genomic DNA was determined by performing high-resolution mass spectrometry. Each nucleotide in the genomic DNA was isolated by performing large-scale HPLC. The eight fractions are shown in Fig. 6a. The molecular weight of the separated nucleotides was determined by performing LCMS-IT-TOF. The results are shown in Fig. 6b; four types of molecular weights were confirmed among the methylated nucleotides, demonstrating that the types of methylated nucleotides in the genomic DNA included not only m4C or m6A but also mG or mT.

### Analysis of the *C*. *militaris* transcriptome

**Figure 7:**
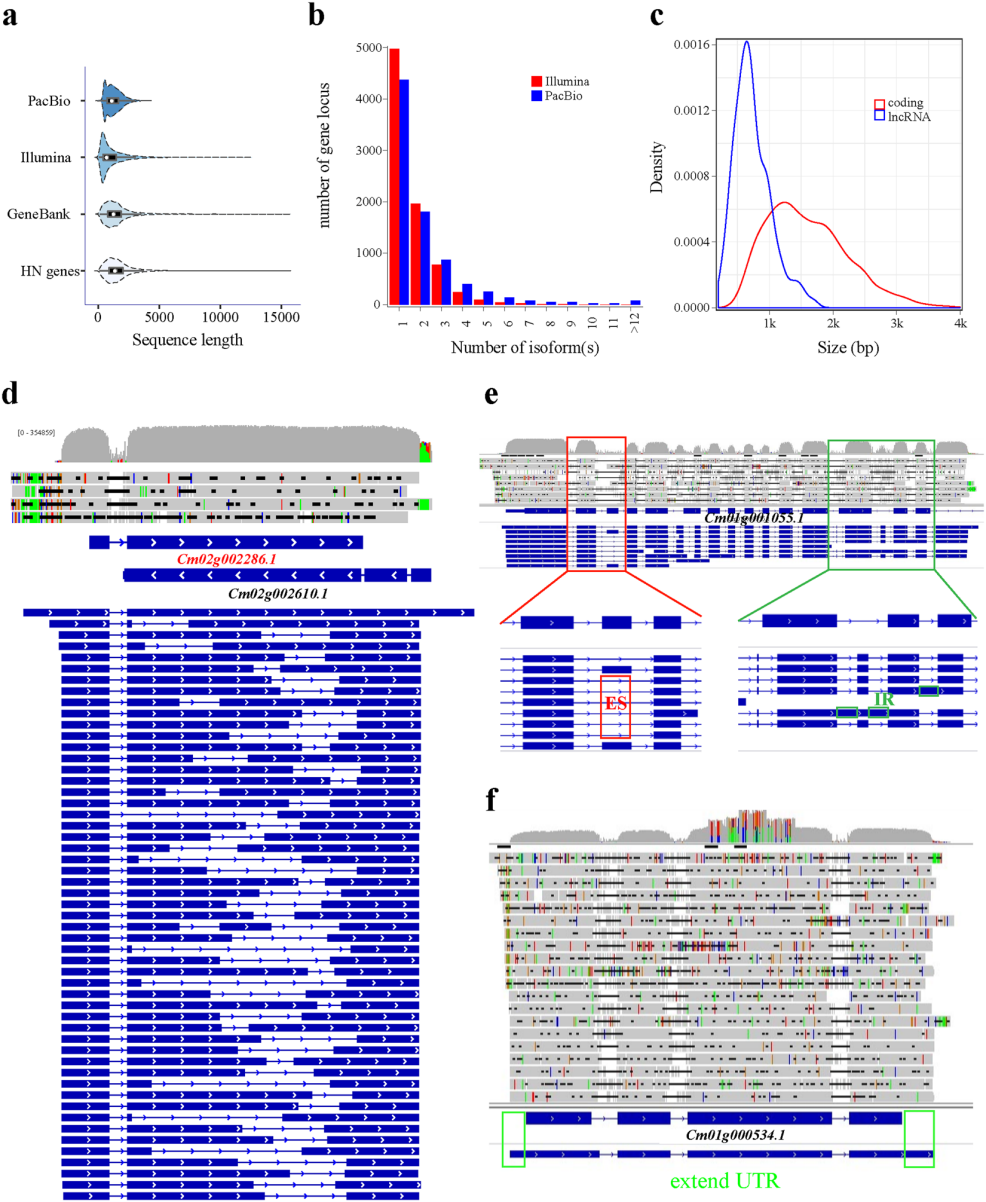
Complexity of the HN fruiting body transcriptome based on PacBio Iso-Seq. (**a**) Comparison of the length of the ToFU transcript set in this study, Illumina short-read assembly transcript set, *C*. *militaris* GenBank reference mRNAs and gene annotation in this study. (**b**) Comparison of the number of isoforms between the Illumina short-read assembly transcript set and the ToFU transcript set. (**c**) Length distributions of the coding and long noncoding ToFU transcript sequences. (**d**) Alignment of the reference annotated transcript (blue) of the Cm02g002286.1 gene with 35 distinct PacBio isoforms. (**e**) Visualization of the alternative splicing of the Cm01g001055.1 gene; the exon-skip (ES) and intron-retain (IR) AS are highlighted. (**f**) Visualization of the extended UTR of the Cm01g000354.1 gene.

**Figure 8:**
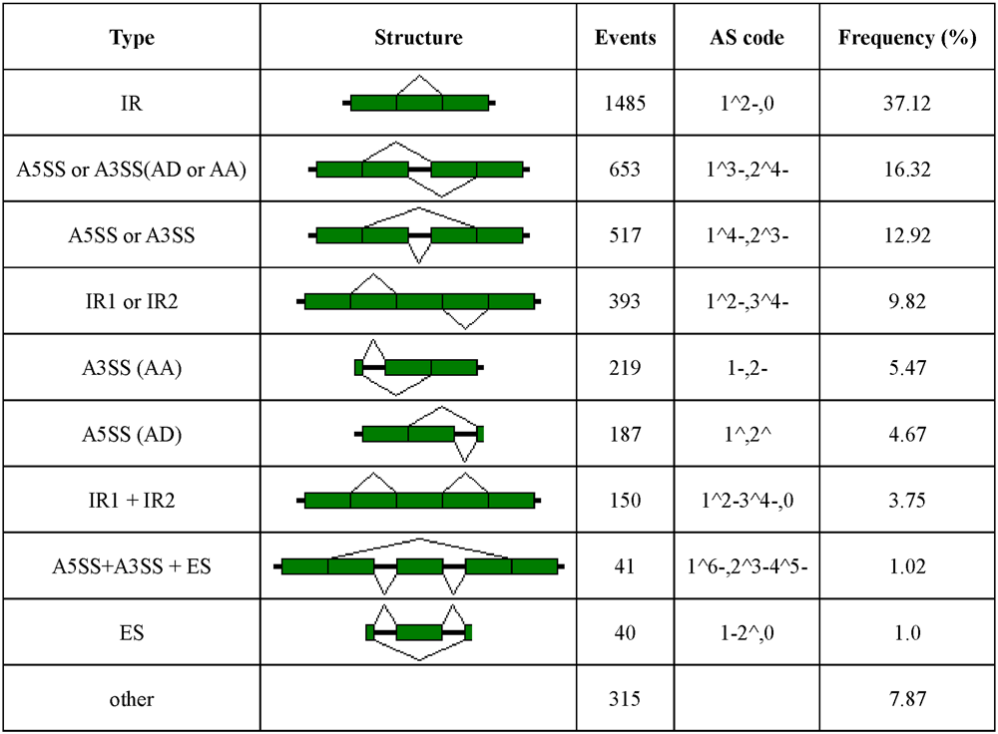
Distribution of mRNA alternative splicing events in the HN strain detected in the PacBio Iso-Seq full-length transcripts.

We performed the initial data processing using a SMRT analysis 2.3.0 Iso-Seq pipeline. From 5 SMRT cells, we produced 5.39 Gb of raw data, with mean read length of insert 1,037 bp to 1,814 bp (Supplement 2). The Iso-Seq pipeline produced 42.0 Mb of polished high-quality consensus isoforms and 26.2 Mb of polished low-quality consensus isoforms. The high-quality consensus isoforms, which covered 8,132 gene loci with 3,756 loci, had more than two isoforms, a maximum length of 5,889 bp, a median length of 1,176 bp, a mean length of 1,275 bp, an N50 length of 1,520 bp and a total number of 31,133 transcripts. BUSCO analysis showed that the transcriptome covered 1,030 (78.3%) of the universal orthologs in Ascomycota, indicating that many genes were silenced in the fruiting stage. In contrast to Illumina RNA-Seq, PacBio Iso-Seq does not require assembly to obtain the full-length transcripts; thus, the errors caused by the short-read assembly are reduced and the integrity and reliability of the transcriptome are improved. A violin plot was generated to show the size of the fruit body. The PacBio set of full-length transcripts was between 350 bp and 2,500 bp (Fig. 7a). Compared with the Illumina RNA-Seq set, the PacBio Iso-Seq set produced more isoforms with additional splicing gene loci. This advantage of PacBio Iso-Seq allows for the direct generation of full-length transcripts and avoids the misassembly of multiple similar isoforms into one transcript. For example, the Cm02g002286.1 gene has an antisense transcript (Cm02g002610.1) that was annotated to produce a single transcript but was found to generate 35 splice variants, as shown in Fig. 7b,d. In addition, 355 lncRNAs with two or more exons and larger than 300 bp were identified and compared with coding transcripts that exhibited shorter sequences (Fig. 7c). Alternative splicing (AS) plays a crucial role in fungal development as well as stress responses; however, alternative splicing events in *C*. *militaris* are poorly understood. Both IR and ES events were identified in the Cm01g001055.1 gene (Fig. 7e). Additionally, untranslated regions (URT) were extended by PacBio Iso-Seq (Fig. 7f), resulting in 4,418 (43.8%) genes with either an extended 5′-UTR or 3′-UTR and 2,309 (22.9%) genes with both UTRs extended. We detected 4,000 AS events from the Iso-Seq reads (Figs. 8), and 1,337 gene loci were involved in the AS events. Intron-retain (IR) events occurred in 3.127% (1,485/4,000) of the reads and were the most frequent AS events in *C*. *militari*s, whereas only 40 exon-skip events (ES) were detected. We also identified 67 potential polycistronic transcripts, including 61 gene loci involved in read-through transcripts. Protein-coding mRNAs with general functions (class R) are the most abundant protein-coding mRNAs, and their number approached 3,000, accounting for 28.7% of all predicted proteins identified using KOG annotation (Supplement 3). The pyrimidine metabolic pathway in the *C*. *militaris* fruiting body is shown in Supplement 4. These proteins are all involved in house-keeping functions in the fungus. In addition, 632 proteins were related to the biosynthesis, transport and catabolism of secondary metabolites. Approximately 25% (2,490/10,095) of the genes were annotated in the KEGG database^22^ and were distributed in 66 pathways. Of these genes, 769 genes were involved in metabolic pathways, 106 genes were involved in carbon metabolism, 98 genes were involved with ribosomal proteins and 98 genes were involved in RNA transport (Supplement 5).

### Structure of the nontranscribed regions

**Figure 9:**
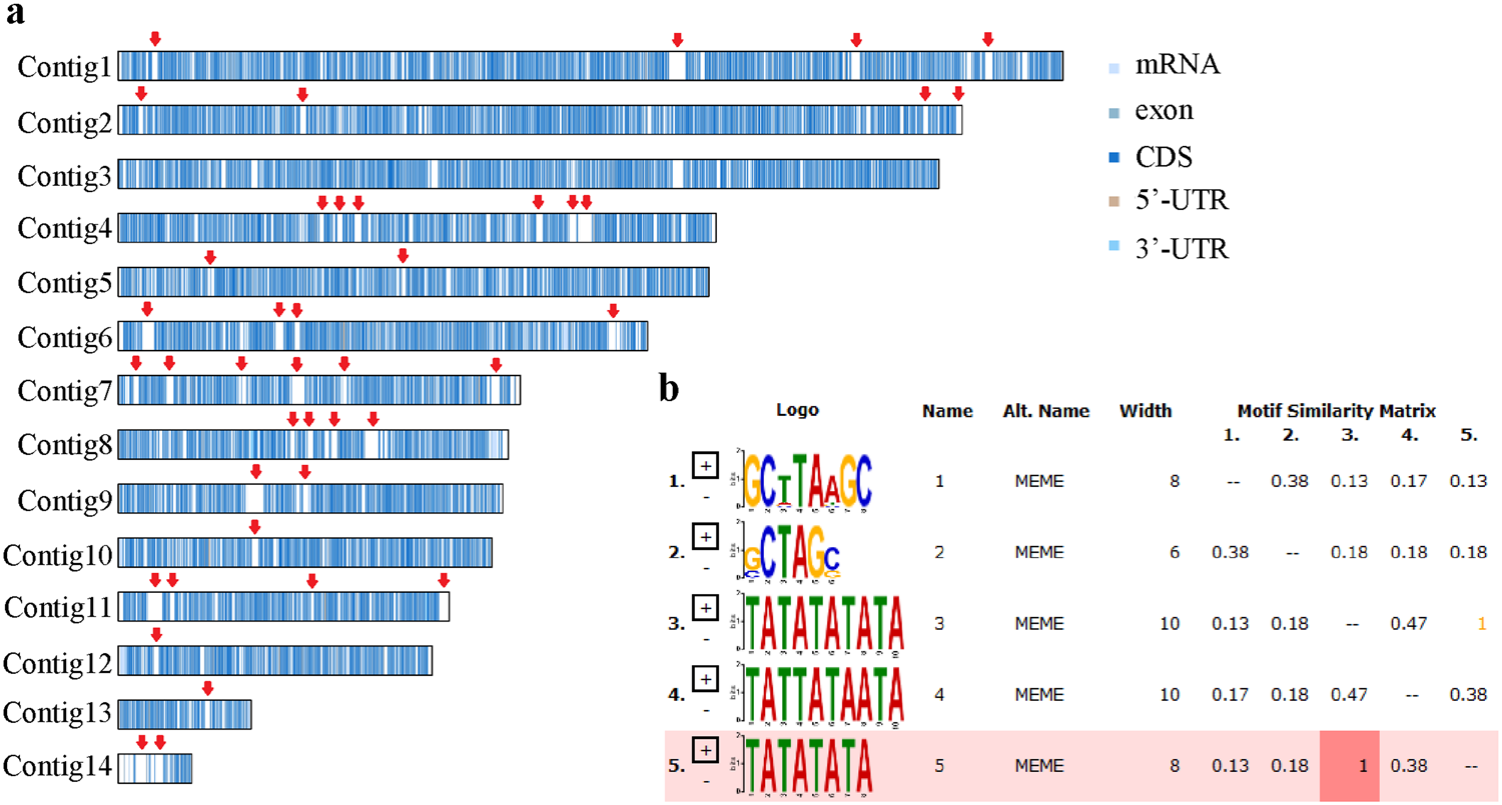
Distributions of the transcriptome and nontranscriptome in the genome of the HN fruiting body. Top 5 motifs in the palindrome structure of the conserved sequence in the nontranscribed region. (**a**) HN fruiting body genomic nontranscribed region. (**b**) Conserved sequence.

The distribution of the transcribed genes in the fruiting body is shown in Fig. 9a. In total, 6,881 nontranscribed regions were identified with an average length of 2.7 kb; the longest region was 80.7 kb. Of the nontranscribed regions, 182 regions were 5–10 kb and 18 regions had >10 kb repetitive sequences with >90% homology. Of the >10 kb homologous fragments, most fragments were mainly adjacent to the two ends of the contigs, whereas the 5–10 kb repeats were distributed throughout each contig. Further analysis of the >50 kb nontranscribed regions among the 6 contigs identified seven regions larger than 7 kb that were homologous repeats. Two repeats were located in contig 1, and the remaining six repeats were distributed in contigs 4, 6, 8, 9 and 11. In addition, 9 homologous sequences (>10 kb) existed within the <50 kb nontranscribed regions. An alignment of these 16 repeats indicated that 71.1% of the sequences were conserved and were AT-rich (>87%). A more detailed analysis showed that the structure of the repeats was palindromic (Fig. 9b). We also found 5–8 bp TATA motifs within those regions; the sequences and frequencies of the top 5 motifs are shown in Fig. 9b.

### Structural variants in the HN strain compared with the CM01 and ATCC 34164 strains

**Figure 10:**
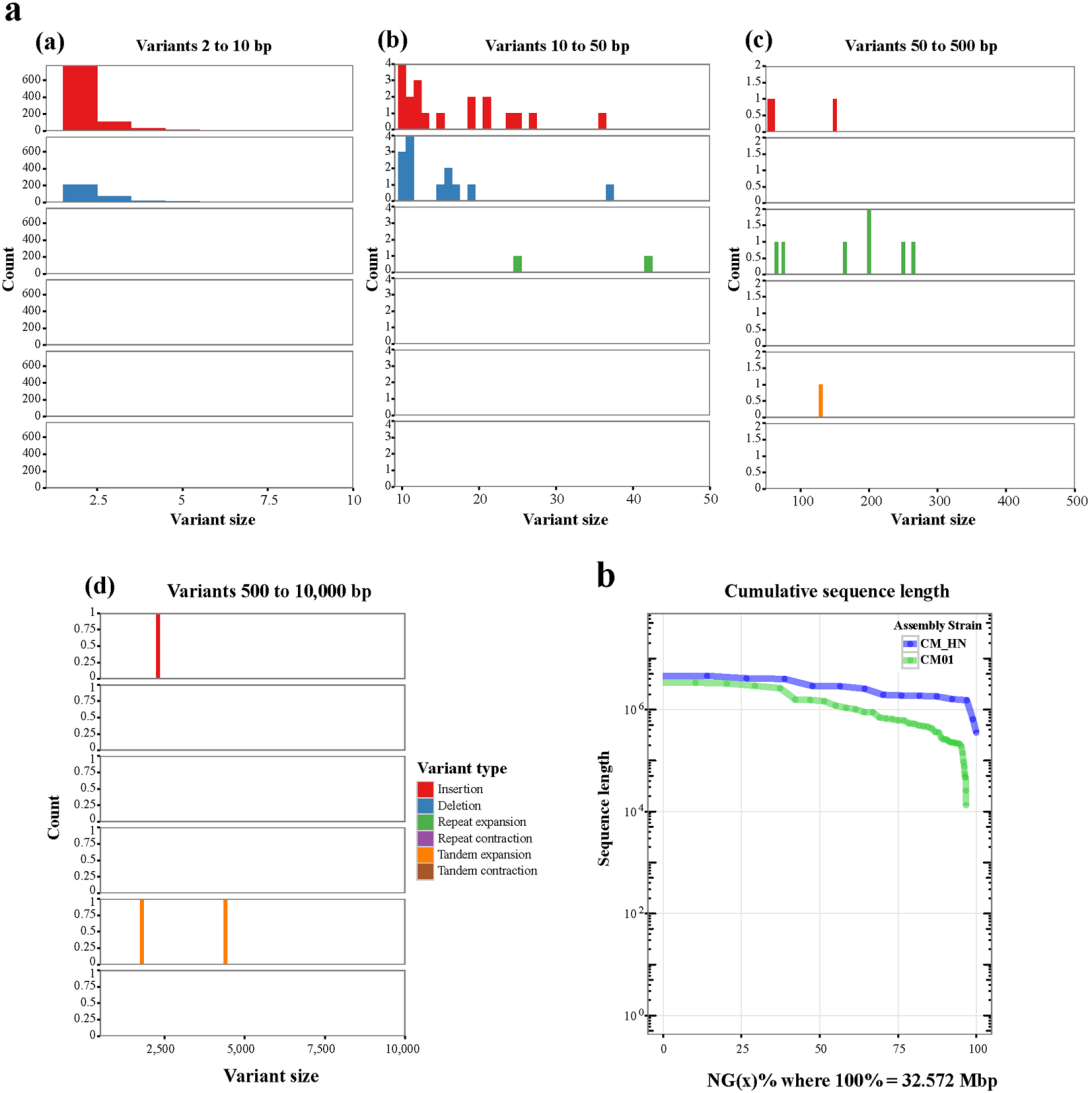
Most variations in the SMRT assembly relative to CM01 are small insertions. Variants ranging from 2 bp to 10 kb in size were called using Assemblytics. (**a**) Size distribution analysis of variants from 2 bp to 10 kb in size; the x-axis represents the variant size in base-pairs and the y-axis represents the variant number. (**a**) Variants from 2 to 10 bp (**b**). Variants from 10 to 50 bp (**c**). Variants from 50 to 500 bp (**d**). Variants from 500 bp to 10 kb. (**b**) Cumulative sequence length plot showing the nearly identical contiguity and total size of the SMRT assembly (query; in green) versus the reference (in blue). The length of each individual sequence is indicated on the y-axis and the cumulative sum of the sorted sequence lengths is indicated on the x-axis.

To examine the genetic variations between the HN and CM01 strains, whole-genome alignment was performed using MUMMER^23^, and many structural variants (SV) were identified according to an assembly based on the SV detection tool Assemblytics^24^. As summarized in Table 3, we identified 1761 insertions, 561 deletions, 8 tandem expansions, 19 tandem contractions, 77 repeat expansions and 215 repeat contractions ranging from 2 bp to 10 kb between the HN and CM01 strains; the size distribution of these structural variations is depicted in Fig. 10. The SV between the HN and ATCC 34164 strains was also examined and 22,158 insertions, 21,130 deletions, 3 tandem expansions, 8 tandem contractions, 322 repeat expansions and 301 repeat contractions ranging from 2 bp to 10 kb were identified. Additionally, 21,885 insertions, 22,408 deletions, 5 tandem expansions, 4 tandem contractions, 336 repeat expansions and 454 repeat contractions, were identified between the CM01 and ATCC 34,162 strains.

**Table 3:** Size distribution of the structural variants in the SMRT assembly relative to the CM01 genome.

Comparing objects	Size range (bp)	Variant type											
		Insertion		Deletion		Tandem_expansion		Tandem_contraction		Repeat_expansion		Repeat_contraction	
		Count	Total (bp)	Count	Total (bp)	Count	Total (bp)	Count	Total (bp)	Count	Total (bp)	Count	Total (bp)
HN_vs_CM01	2–10	1551	3545	533	1628	0	0	0	0	12	67	18	86
	10–50	17	292	25	382	0	0	0	0	43	1159	36	1030
	50–500	191	18181	2	361	8	1384	19	3202	20	3517	114	19484
	500–10,000	2	1989	1	1587	0	0	0	0	2	1194	47	60505
	Total	1761	24007	561	3958	8	1384	19	3202	77	5937	215	81105
HN_vs_ATCC	2–10	18915	74099	18027	71902	0	0	0	0	14	91	18	90
	10–50	2937	48659	2830	46906	0	0	1	27	29	754	37	1091
	50–500	201	34403	197	26993	3	552	7	1534	120	22934	132	24858
	500–10,000	105	367150	76	266652	0	0	0	0	159	530194	114	395766
	Total	22158	524311	21130	412453	3	552	8	1561	322	553973	301	421805
ATCC_vs_CM01	2–10	18624	74100	19135	75435	0	0	0	0	25	134	16	94
	10–50	2874	47367	2995	49499	1	26	0	0	62	1722	64	1715
	50–500	302	38634	189	32080	4	653	4	514	144	25866	176	32966
	500–10,000	85	284457	89	301499	0	0	0	0	105	349498	198	661564
	Total	21885	444558	22408	458513	5	679	4	514	336	377220	454	696339

## Discussion

We used SMRT sequencing technology to assemble the complete genome of the *C*. *militaris* HN strain, which is 32.57 Mb in size with 14 chromosomes, at the chromosome level, significantly improving our knowledge of the genome.

The genome of the ATCC 34164 strain of *C*. *militaris*, a strain isolated from butterfly pupae, has 7 contigs, four of which have telomeric repeats (GGTAA or TTAGGG) on either the 5′ or 3′ end of the contig^14^. The genome of *Cordyceps guangdongensis* has 9 scaffolds and a genome size of 29.05 Mb^25^. The haploid genomes of *C*. *militaris* and *Cordyceps subsessilis* both contain seven chromosomes. However, in our study, 4 contigs had telomeric sequences on both ends and the other 8 contigs had telomeric sequences on the 5′ or 3′ end, suggesting that the actual number of chromosomes in *C*. *militaris* needs to be further verified by karyotype analysis. These three public strains were isolated from different insect hosts, and they vary in the number of repeats, the GC content, and gene numbers, providing us with valuable resources for a fungi-insect host interaction and relationship study.

The genome of the *C*. *militaris* HN strain was determined to have both MAT 1–1–1 and MAT 1-1-2 mating-type genes on contig 3, while there were no MAT 1-2-1 mating-type genes in our present assembled genome and raw subreads, supporting the notion that *C*. *militaris* is heterothallic (Supplement 6). A previous study showed that both the MAT 1-1- and MAT 1-2-containing isolates are able to fruit. The materials used for genome sequencing may have come from asexual fruiting bodies and are consistent with a relatively low heterozygosity rate by GenomeScope analysis^26^ (Supplement 7).

We obtained 31,133 high-quality transcripts, which covered 8,132 gene loci, with 3,756 loci having more than two isoforms. In contrast, a previous study showed that 9,010 genes can be mapped in the fruiting body by Illumina RNA-Seq^27^. The 878 genes that could not be mapped will be studied in the future, and the two technologies will be compared. AS is an important mechanism for regulating gene expression and generating proteome diversity^27–29^. In this study, 1,337 (13.2%) genes associated with AS were detected in the fruiting body, while 368 (3.6%) genes in the same tissue were detected by Illumina RNA-Seq, suggesting that Iso-Seq may increase the number of AS events that are detected. The AS rate of *C*. *militaris* was much lower than those of animals and plants; these results are similar to those of a previous study in *Fusarium graminearum*^30^. Furthermore, 352 AS genes were annotated with KEGG pathway information. These results suggest that stage-specific AS genes might have important functions in fungi development. Widespread polycistronic transcripts in several *Agaricomycetes* were identified by SMART Iso-Seq^31^, involving up to 8% of the transcribed genes. In our study, 67 potential polycistronic transcripts, including 61 gene loci that were involved in read-through transcripts, were discovered. However, the function of these polycistronic transcripts requires further experimental characterization. This finding suggests that polycistronic transcripts may be a conserved feature throughout the fungal transcriptomes.

Using the genome and transcriptome data, we obtained the complete, high-quality nontranscribed region. The longest region in the nontranscribed region can reach over 80 kb. By analyzing the structural features of the DNA in the nontranscribed regions, 5–8 bp TATA motifs within these regions were found. TATA-box and Initiator (*Inr*) elements are two main key cis-regulatory elements within a core promoter^32^, suggesting that nontranscribed regions are the starting regions of genomic DNA replication and may function as regulatory elements to control gene expression. These regions exhibit the structural characteristic of having high AT content; thus, the double helix structure of the DNA can be easily opened^33^.

A genome-wide methylation map was constructed using SMRT. The methylation characteristics of *C*. *militaris* were mainly in the form of m6A and m4C, with methylation rates of 0.0164% and 0.0846%, respectively. In addition, many other DNA modification patterns were observed in the genome at a modification rate of 2.0017%. However, previous reports indicated that in fungi that have genomic 5-methylcytosine (m5C), only repetitive DNA sequences are methylated^34^. Therefore, many unknown forms of DNA modification remain to be explored. This difference may be due to variations in sequencing technologies, and it is worthwhile for us to discover new forms of methylated nucleotides.

In 1980, HPLC was used to detect and analyze methylation levels in DNA samples^35^. To detect and analyze DNA methylation in depth, we obtained a sufficient quantity of genomic DNA from *C*. *militaris* by performing a large-scale extraction, and then, many single nucleotides were prepared using large-scale separations. Using high-resolution LC-MS to analyze the molecular weights of the four nucleotides in the *C*. *militaris* genome, we discovered that four types of nucleotide methylation existed in the genomic DNA, especially the methylation of thymine, which proved its existence for the first time. Thus, all four nucleotides were likely methylated in the genomic DNA from *C*. *militaris*. This result may provide favorable evidence and new ideas for studying genomic DNA modifications. It also provides indirect evidence that supports the existence of a large number of unknown DNA modifications based on the PacBio methylation assay.

Large-scale interchromosomal translocation events were detected in the whole-genome alignments among the paired genomes of the HN, CM01 and ATCC strains. An in-depth investigation of the translocation breakpoint revealed transposable elements (TEs) and the composition of the flanking sequence of the translocation breakpoint, suggesting that TEs play a crucial role in driving genomic plasticity. In total, 2,816 structural variants were identified using an assembly-based SV detection tool. The translocation and structural variants identified herein contributed significantly to our understanding of the complexity of insect-pathogenic fungus biology and the biosynthesis pathway of pharmacologically active compounds.

In conclusion, our study provides genome, transcriptome and methylome data for a new strain of *C*. *militaris*, paving the way for research that comprehensively assesses genetic variation at all size scales and methylation at a single-base resolution. The methylation motifs of m6A and m4C in the genome of the HN strain of *C*. *militaris* were analyzed, and the four methylated nucleotides were identified. Through the transcriptome obtained from Iso-Seq, many unknown RNA splicing patterns were discovered. At the same time, there are many conserved TATA-box structures in the nontranscribed regions of the genome. The results will provide a basis for further research on the molecular biology of fungi.

## Methods

### Fungus strain and maintenance

The *C*. *militaris* strain Haining (HN) was isolated from a single spore by Zhejiang Chinagene Biomedical Co. Ltd and was identified by the Institute of Microbiology Chinese Academy of Sciences^36^. The culture was maintained on either artificial medium or silkworm pupae at 23 °C. *C*. *militaris* was cultured for 90 days in our laboratory, and the fruiting bodies were used for the extraction of the genomic DNA and total RNA.

### Genomic DNA extraction

The *C*. *militaris* genomic DNA was extracted using the sodium dodecyl sulfate (SDS)-phenol method. First, the *C*. *militaris* fruiting body was lysed with 3% SDS (0.1 M Tris-HCl (pH 8.0), 0.5 M NaCl, 0.05 M EDTA, 3% SDS) and proteinase K at a final concentration of 50 μg/ml was added to the mixture, which was incubated at 65 °C for 12 hours. After centrifugation at 10,000 rpm for 10 min, the supernatant was extracted three times with an equal volume of 0.1 M Tris-phenol (pH > 7.5). The flocculated DNA was obtained by adding 2.5 volumes of ethanol to the supernatant at 4 °C for 30 min after centrifugation at 10,000 rpm for 10 min, and then, the DNA was dissolved in H_2_O and digested with RNase A for 30 min; the solution was re-precipitated with 70% ethanol. Finally, the DNA was purified using a PowerClean DNA cleanup kit (MoBio, Carlsbad, CA). The quality of the extracted DNA was checked using 0.7% agarose gel electrophoresis and was determined using a NanoDrop spectrophotometer and quantified using Qubit (Thermo Fisher Scientific). The extracted DNA was stored at −80 °C until further analysis.

### DNA library preparation and sequencing

A large-insert PacBio library was prepared using a SMRTbell™ Template Prep Kit version 1.0 (Pacific Biosciences) according to the manufacturer’s instructions. In brief, the fungal DNA was sheared to a targeted size of approximately 20 kb using g-TUBEs (Covaris, Inc., USA). The sheared genomic DNA was subjected to DNA damage repair/end repair and blunt-end adaptor ligation, followed by exonuclease digestion. The purified digestion products were loaded onto pre-cast 0.6% agarose gels for a 7–50 kb size selection using a BluePippin Size Selection System (Sage Science), and the recovered size-selected library products were purified using 0.5× pre-washed PB AMPure beads (Beckman Coulter). The library concentration was determined using a Qubit 2.0 Fluorometer (Life Technologies). The libraries were sequenced using P6C4 polymerase and chemistry on a PacBio RS II instrument with 240 min movie times at Tianjin Lakeside Powergene Science Development Co. Ltd. (Tianjin, China). In total, 13 SMRT Cells were used to yield 10.8 Gbp.

### Total RNA extraction, Iso-Seq library preparation and PacBio sequencing

Total RNA was isolated using a UNIQ-10 column TRIzol total RNA extraction kit (Sangon Biotech) according to the manufacturer’s instructions, followed by treatment with DNase I. The mRNA was purified by a poly T column separation and stored at −80 °C until further analysis. The Iso-Seq library was prepared according to the PacBio Isoform Sequencing protocol (Iso-Seq™). The RNA was reverse transcribed using a SMARTer® PCR cDNA Synthesis Kit and was PCR amplified using KAPA HiFi PCR Kits. These cDNA products were purified using a SMRTbell DNA Template Prep Kit 3.0 for library construction. The libraries were sequenced using P6C4 polymerase and chemistry on a PacBio RS II platform with 240 min movie times at Tianjin Lakeside Powergene Science Development Co. Ltd. In total, 7 SMRT Cells were used to generate 4.4 Gbp of transcriptome cDNA sequencing data.

### *De novo* genome assembly

The *de novo* assembly of the whole *C*. *militaris* genome was performed using the RS_HGAP_Assembly.3 protocol implemented in SMRT Analysis Portal 2.3.0.p5^6^ (Supplement 8). All parameters were set to the default settings with the following exceptions: subread length = 9,000; minimum seed read length = 11,000; genome size 35,000,000; and target coverage = 30. The filtered reads were mapped to the contigs using Blasr^37^ and the contigs were polished using Quiver^6^ to generate a high-quality genome and then visualized using the Integrative Genomics Viewer (IGV)^38^.

### Repeat and noncoding RNA annotation

The telomeric repeats and tandem repeats were identified using Tandem Repeat Finder (v. 4.07b)^39^. Known transposable element repeats were annotated using RepeatMasker (v. 4.0.7) and RepeatProteinMasker^40^ to search against the Repbase library (Repbase Library 20150807)^41^. The *de novo* transposable element prediction was performed using RepeatScout (version 1.0.5)^40^. The combined results generated the comprehensive *C*. *militaris* TE database. The noncoding RNA, including rRNA and tRNA, were predicted using rRNAmmer 1.2^42^ and tRNAscan1.23^43^.

### Gene prediction and functional annotation

The gene prediction was performed using the MAKER (version 2.31.8) pipeline. All RefSeq protein sequences in *Hypocreomycetidae* were downloaded from GenBank and used as protein evidence in MAKER. The EST sequence from *C*. *militaris* and the high-quality Iso-Seq full-length CDS set were combined and used as EST evidence. First, we used Augustus, trained for *Fusarium graminearum*, and GeneMark-ES and SNAP, trained for *Caenorhabditis elegans*, for the *ab initio* gene prediction. Based on these MAKER results, we trained the Augustus and SNAP gene prediction model. Next, MAKER was run using the in-house training Augustus and SNAP parameters, and a gene set was generated as the gene models of the *C*. *militaris* genome. The gene models were functionally annotated using the NCBI nonredundant (NR), UniProt^44^, GO, COG, and KEGG^22^ databases. Matches with an e-value <1e-5 and >40% sequence identity were selected. The gene families were established using the Interpro database using BlastProDOM, HMMPIR, HMMPfam, SuperFamily, SignalPHMM, and HMMPanther^45^. The secondary metabolite genes and gene clusters were predicted using both AntiSMASH, fungal version 4.0.0 and SMURF (accessed June 2017)^46,47^.

### Iso-Seq data analysis

The standard RS_IsoSeq. 1 protocol (SMRT Analysis 2.3.0p5) was used to process the raw sequencing data. In summary, the ROIs were generated and separated into full-length and non-full-length ROIs using ‘pbtranscript.py classify’. The full-length ROIs were clustered and assembled into consensus sequences by performing isoform-level clustering using an ICE algorithm with estimated cDNA sizes between 1–2 kb. Subsequently, the consensus sequences were polished based on the non-full-length ROIs and categorized as HQ (above 99% accuracy) or LQ full-length polished consensus transcripts using Quiver. All high-quality (HQ) transcripts were mapped to the *C*. *militaris* genome using GMAP with the parameters ‘–cross-species -B 5 -K 8000 -t 40 -f 2 -n 1’ and filtered for a >99% alignment coverage and >85% alignment identity^48^. The above GFF3 format was transferred into the GTF format using an in-house python script. Then, the alternative splicing (AS) events were identified based on the above GTF file using the ASTALAVISTA algorithm^49^. High-quality (HQ) transcripts that could not be aligned were considered novel transcripts. The long noncoding RNAs (lncRNAs) were identified as described in our previous study^50^. The genome‐wide detection of base modifications was performed using the “RS_Modification_and_Motif_Analysis.1” protocol (SMRT Analysis 2.3.0p5 with the default parameter settings; the *C*. *militaris* genome was used as a reference, and only unambiguously mapped reads were used for the base modification detection. Then, we further filtered the modified sites with a less than 50× coverage and a quality value (QV) score less than 20. For each m6A and m4C, we extracted 2 bp from the upstream and downstream sequences. MEME-ChIP^51^ was used to identify the motifs in each group.

### LC-MS analysis of base methylation types (m6A and m4C)

Based on the approximately 0.1% methylation rate in the genome, we used single-clone HN 30 kg to extract the genomic DNA. In total, 30 g of genomic DNA were obtained. The DNA was digested by DNase P1. Then, we used Agela’s FLEXA HPLC purification system with a chromatographic column as follows: X-AMIDE, 10.0 × 250 mm; and Venusil XBP-C18. The separated products were dried at an ultra-low temperature. The sample was concentrated by a rotary evaporator and dissolved in water. The sample was separated, and the molecular weight was determined using a Shimadzu mass spectrometer (LCMS-IT-TOF). Methylation was identified by comparing the molecular weight with the predicted molecular weights of the methylated four types of nucleotides. The detailed protocols follow.

### Genomic DNA extraction of *C*. *militaris*

Genomic DNA was extracted with 3% SDS. The 4,000 g fruiting bodies were subjected to superfine grinding using an ultralow temperature crusher at −80 °C. We added 20 L of DNA extraction buffer (0.1 M Tris HCl (pH 8.0), 0.5 M NaCl and 0.05 M EDTA, 3% SDS) and 50 µg/ml Protease K (20 mg/ml) and digested the mixture overnight at 65 °C. Isovolumetric phenol (0.1 M Tris saturated phenol, pH > 7.5) was used three times at 10,000 rpm for 10 min for the extraction. An equal volume of chloroform:isoamyl alcohol (24:1) was used twice at 10,000 rpm for 10 min for the extraction. We added 2.5 times the volume of anhydrous ethanol precipitate and mixed it well with a cryogenic static >30 min. After centrifugation at 10,000 rpm for 8 min, the precipitate was collected, washed 3 times with 75% ethanol, dried in ethanol at 20 °C and resuspended in water. The sample was checked by 0.7% agar gel electrophoresis.

### Preparation of genomic DNA

We added RNase A (10 mg/ml) to a final concentration of 100 µg/ml at 37 °C and incubated for 1 hour. Isovolumetric phenol (0.1 M Tris-saturated phenol, pH > 7.5) was used at 10,000 rpm for 10 min for the extraction. An equal volume of chloroform:isoamyl alcohol (24:1) was used at 10,000 rpm for 10 min for extraction. The supernatant was collected, and we added 2.5 times the volume of ethanol for the precipitation, which occurred at −20 °C for 30 min. The sample was then centrifuged at 10,000 rpm for 8 min, and the centrifugal sedimentation was used to obtain the genomic DNA, while the supernatant was used to obtain the RNA degradation products. The sample was subjected to centrifugal precipitation with 75% ethanol, washed 3 times, blown dry, suspended in water and stored at −20 °C. The sample quality was checked using 0.7% agar gel electrophoresis.

### Ultrasonication and digestion of heat-denatured DNA with DNase P1

We added the DNA to the ultrasonic cell disrupter and applied ultrasonication three times for 3 seconds. The DNA solution was adjusted to a pH of 6.5 with hydrochloric acid; then, we added ZnSO_4_ to a final concentration of 2 mM in a water bath at 100 °C for 2 min and transferred the sample to a 70 °C-water bath. We incubated the sample with 20–30% (w/w) DNase P1 for 5 hours. We performed HPLC to determine whether the reaction had reached completeness. After the reaction was complete, we added EDTA-2Na to a final concentration of 10 mM to inactivate the enzyme.

### Separation of DNA degradation products using the Agela FLEXA purification system and detection using a Shimadzu mass spectrometer LCMS-IT-TOF

Purification by chromatography was performed using the following: Column: X-AMIDE, 10 × 250 mm; Phase A: 0.2% acetic acid; Phase B: acetonitrile; Flow rate: 4 mL/min; UV detection wavelength: 260 nm; Sample loading: 1 mL (1 mg/mL); Elution conditions: 5% A to 23% A for 15 min; 23% A to 26% A for 5 min; 26% A to 29% A for 5 min; 29% A to 32% A for 5 min; A, 5 min; and 35% A ~ 40% A, 5 min. The separated products were concentrated in an ultra-low temperature dryer and dissolved in water. The nucleotide molecular weights were identified using a Shimadzu mass spectrometer (LCMS-IT-TOF). The MS liquid phase conditions were as follows: Column: ACQUITY UPLC BEH (2.1 × 100 mm, 1.7 µm); UV detection wavelength: 260 nm; Flow rate: 0.3 mL/min; Phase A: 0.1% formic acid; B phase: acetonitrile; Column temperature: 40 °C; Elution conditions: 2% acetonitrile isocratic elution 10 min; and load sample: 1 µL.

### Whole-genome alignment and structural variation analysis

We downloaded the previously released genomes of the *C*. *militaris* CM01 strain (GCF_000225605.1)^9^ and the *C*. *militaris* ATCC 34164 strain (PRJNA323705)^14^ from GenBank. To identify the structural variations between the genomes, we used MUMmer to perform a whole-genome alignment using HN as a reference genome and the downloaded genomes as query genomes. Then, the Assemblytics algorithm was used to identify the structural variations in six classes of variants: insertions, deletions, tandem expansions, tandem contractions, repeat expansions and repeat contractions^24^. Dot plots of the alignments were generated using Gepard v. 1.4^52^. The alignments of the raw SMRT genome reads to the assembled genomes were performed using Blasr; the Iso-Seq reads were aligned using GMAP^48^, and we visualized the structural variations using the Integrative Genomics Viewer (IGV)^38^.

### Statistics and analysis

The Gene Ontology term analysis of the genes with methylation motifs was conducted using the GOseq Bioconductor package^53^. We considered over-represented GO terms with a Benjamini Hochberg FDR adjusted p-value < 0.05 significantly enriched. We performed a KEGG pathway enrichment analysis of the genes with methylation sites using KOBAS 2.0^54^.

## Supplementary information

Supplement information

C. militaris Genomic data

C. militaris DNA methylation data

C.militaris Transcriptomic data

## Data Availability

The genome and transcriptome data from *Cordyceps militaris* by single molecule real time sequencing were deposited into GenBank. The GenBank number of the genome is MQTM00000000.1. The GenBank number of the transcriptome is GEZI00000000.1.
