# Supplementary material for: Study of the whole genome, methylome and transcriptome of Cordyceps militaris
Source: Sci Rep. 2019 Jan 29;9:898. doi: 10.1038/s41598-018-38021-4 (PMC6351555; doi:10.1038/s41598-018-38021-4)

# Study of the whole genome, methylome and transcriptome of *Cordyceps militaris*

Yujiao Chen[1,2,3,4,5+], Yuqian Wu[1,3,4,5,6+], Li Liu[3,5+], Jianhua Feng[3], Tiancheng Zhang[3], Sheng Qin[7], Xingyu Zhao[7], Chaoxia Wang[3], Dongmei Li[3], Wei Han[3], Minghui Shao[3], Ping Zhao[1], Jianfeng Xue[3], Xiaomin Liu[2,4], Hongjie Li[2], Enwei Zhao[2,4], Wen Zhao[3], Xijie Guo[7], Yongfeng Jin[8], Yaming Cao[9], Liwang Cui[3,10], Zeqi Zhou[11], Qingyou Xia[6*], Yaozhou Zhang[1,2,3,4,5,12*], Zihe Rao[12*]

[1]Human Genome Research Center, Tianjin University, Tianjin, 300309, China.

[2]Zheng-Yuan-Tang (Tianjin) Biotechnology Co. Ltd, Tianjin, 300457, China.

[3]Tianjin Lakeside Powergene Science Development Co. Ltd, Tianjin, 300309, China.

[4]Zhejiang Chinagene Biomedicine Co. Ltd, Jiaxing, 314400, China.

[5]Guizhou Gui'an Academy of Precision Medicine Co. Ltd, Gui'an, 561113, China.

[6]State Key Laboratory of Silkworm Genome Biology, Southwest University, Chongqing, 400715, China.

[7]College of Life Sciences, Jiangsu University of Science and Technology, Zhenjiang, 212000, China.

[8]College of Life Science, Zhejiang University, Hangzhou, 310058, China.

[9]Department of Biochemistry and Molecular Biology, China Medical University, Shenyang, 110001, China.

[10]Department of Entomology, Penn State University, PA, 16802, USA.

[11]Dynamiker Biotechnology (Tianjin) Co., Ltd, Tianjin, 300467, China.

[12]Tianjin International Joint Academy of Biomedicine, Tianjin, 300457, China.

[*]Correspondence and requests for materials should be addressed to Yaozhou Zhang (email: zhangyaozhou88@126.com)

[+]These authors contributed equally to this study

**Supplement 1. Dot plots comparing the HN, CM01 and ATCC strains.**

**a**

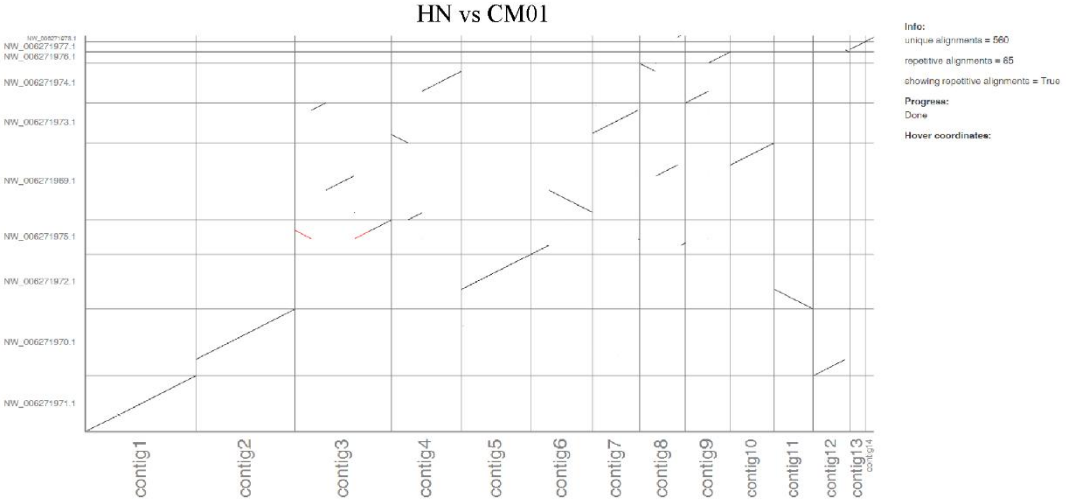


**b**

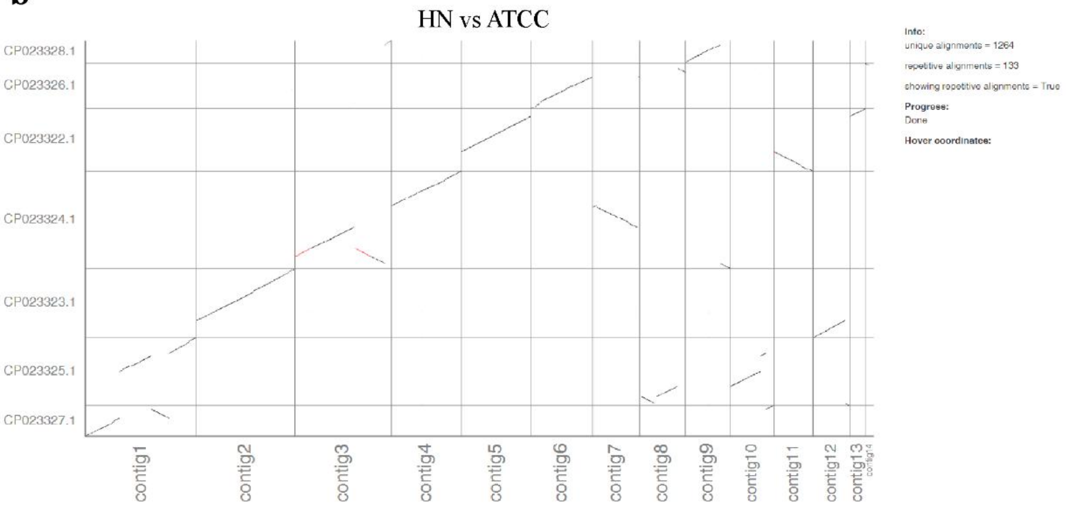


**c**

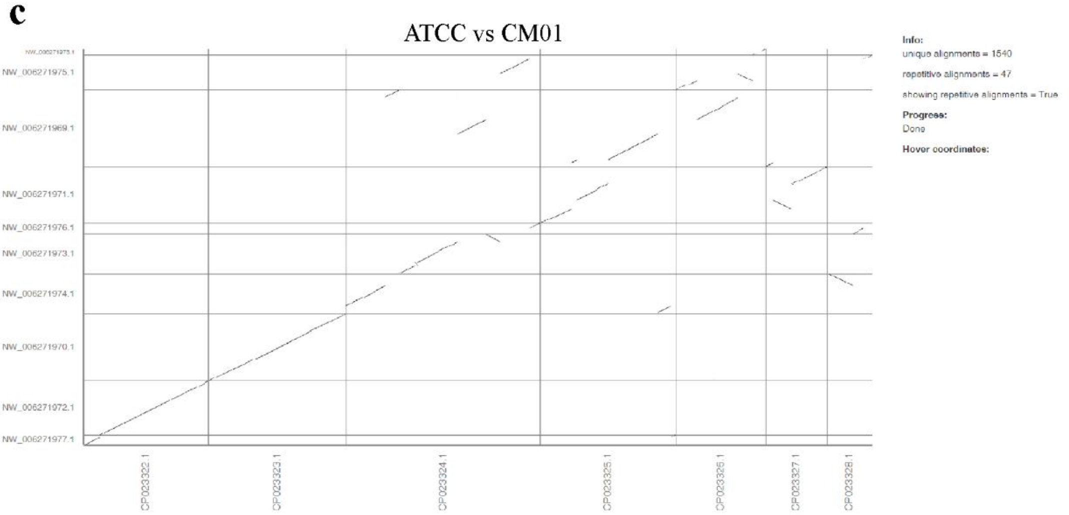

**Supplement 2. Statistics from the Iso-Seq data of the *C. militaris* HN fruiting body.**

| Movie | Reads Of Insert | Read Bases Of Insert | Mean Read Length Of Insert | Mean Read Quality Of Insert | Mean Number Of Passes |
|---|---|---|---|---|---|
| m160131_103047_42235_c100881252550000001823202703261662_s1_X0 | 81728 | 111769443 | 1367 | 0.9402 | 12.06 |
| m160201_093507_42235_c100881252550000001823202703261664_s1_X0 | 66343 | 120407990 | 1814 | 0.9414 | 10.29 |
| m151208_192746_42227_c100886302550000001823202504021601_s1_p0 | 51692 | 67277708 | 1301 | 0.9058 | 8.92 |
| m160201_051416_42235_c100881252550000001823202703261663_s1_X0 | 81007 | 134538427 | 1660 | 0.933 | 10.39 |
| m151203_172940_42234_c100886202550000001823202504021637_s1_p0 | 27493 | 33254687 | 1209 | 0.9103 | 9.58 |
| m151207_054702_42234_c100886142550000001823202504021622_s1_p0 | 9931 | 10306040 | 1037 | 0.8649 | 5.54 |
| m151208_234405_42227_c100886302550000001823202504021602_s1_p0 | 45918 | 61926719 | 1348 | 0.911 | 9.71 |

**Supplement 3. Clusters of orthologous group (COGs) classification in the transcriptome of the HN fruiting body**

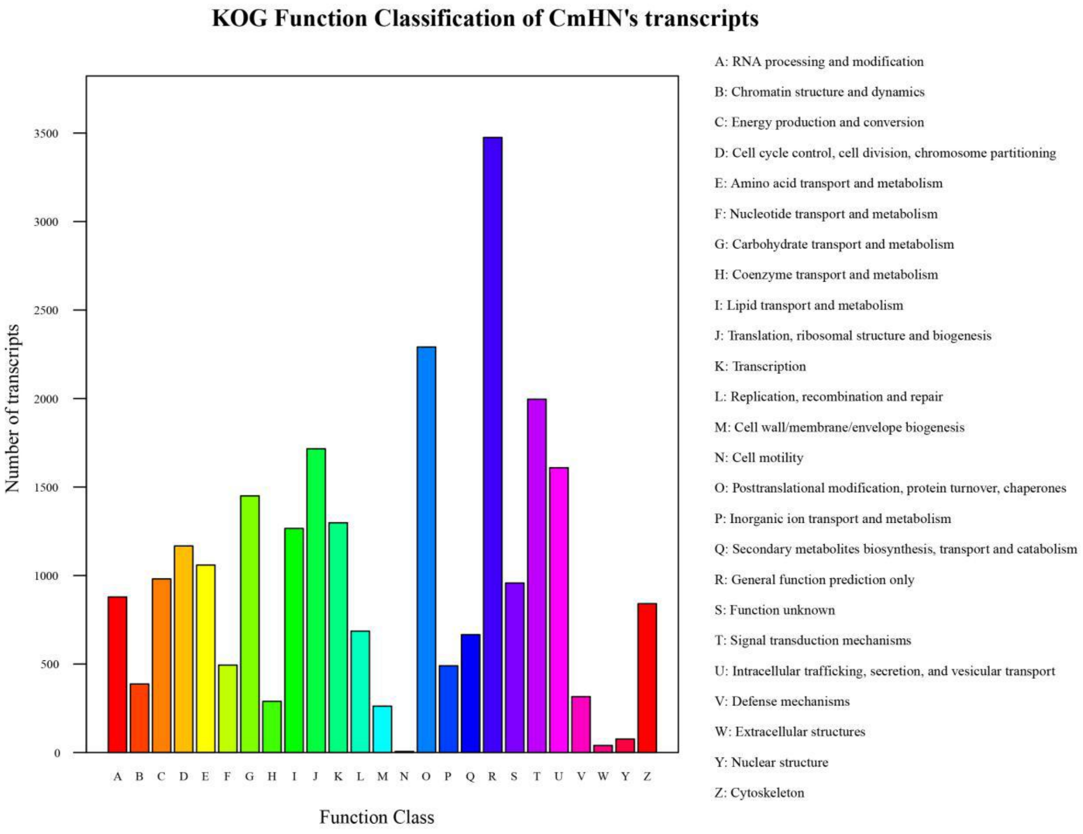

**Supplement 4. Metabolism of pyrimidine in the HN fruiting body.**

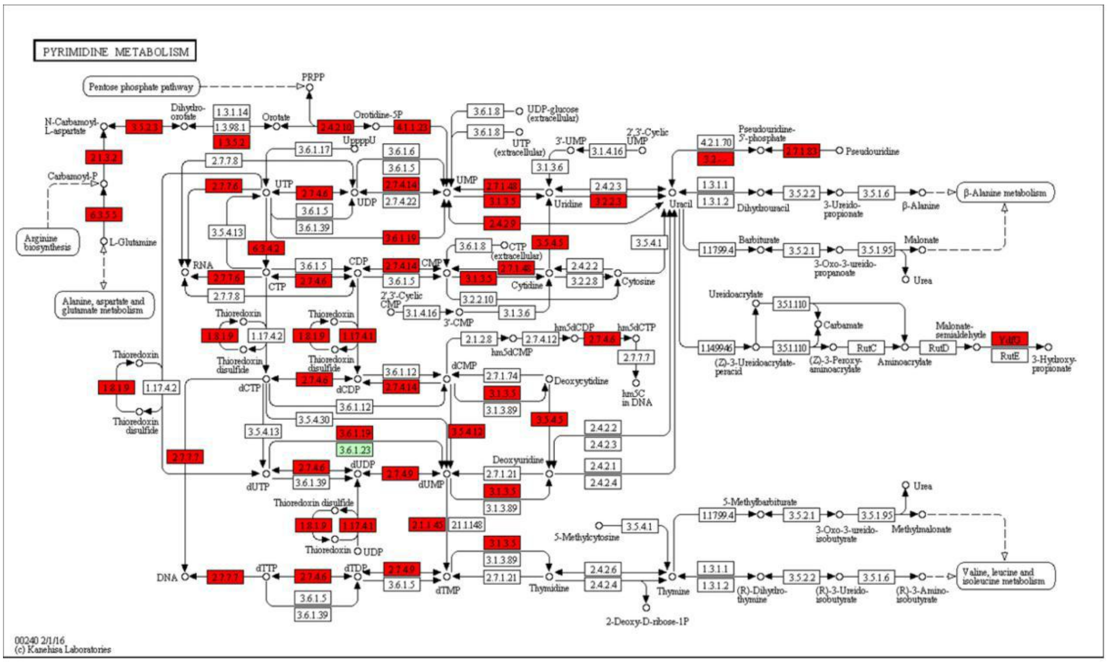


## REFERENCES

Kanehisa, M., Furumichi, M., Tanabe, M., Sato, Y. & Morishima, K. KEGG: new perspectives on genomes, pathways, diseases and drugs. *Nucleic Acids Res.* **45**, D353-D361 (2017).

**Supplement 5. Distribution of the HN fruiting body genes within the KEGG pathways.**

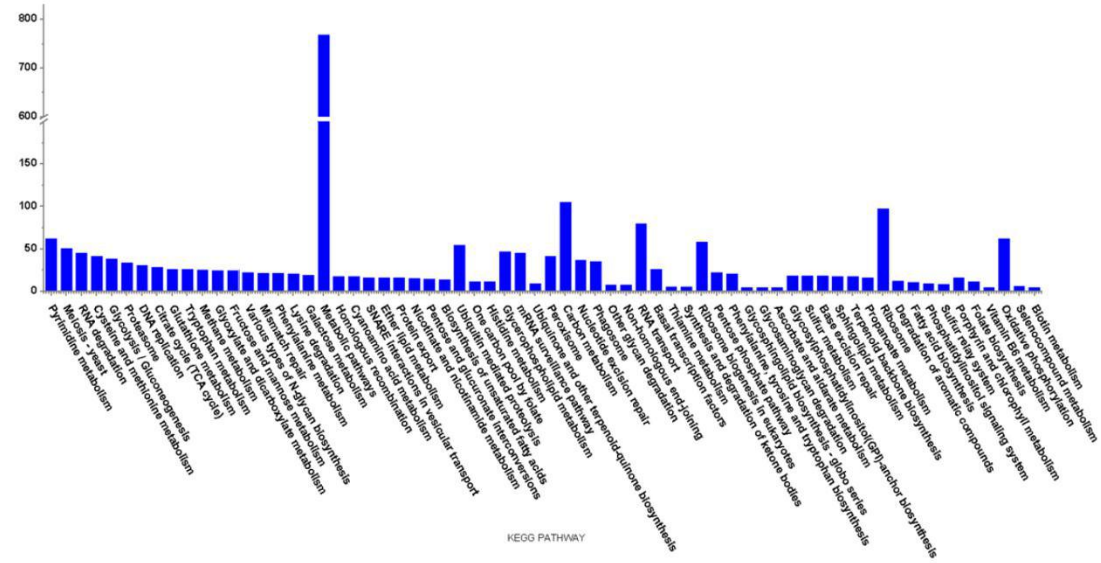

**Supplement 6. Comparison of mating-type loci and surrounding genes in *C. militaris* HN, *C. militaris* ATCC 34164 and *C militaris* CM01.**

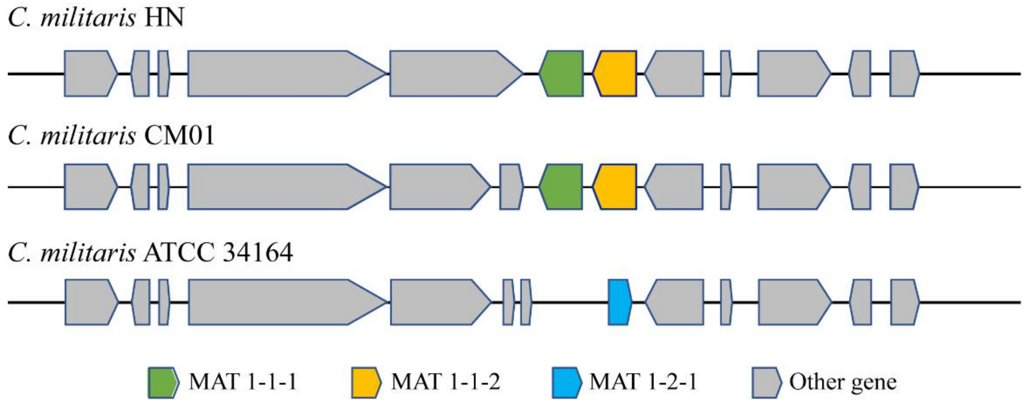

**Supplement 7. Estimation of the genome size, repeat content, and heterozygosity by GenomeScope. a.** GenomeScope k-mer profile plot of *C. militaris* based on 51-mers in error-corrected pacbio reads from CANU. **b.** GenomeScope k-mer profile plot of *C. militaris* based on 71-mers in error-corrected pacbio reads from FALCON.

**a**

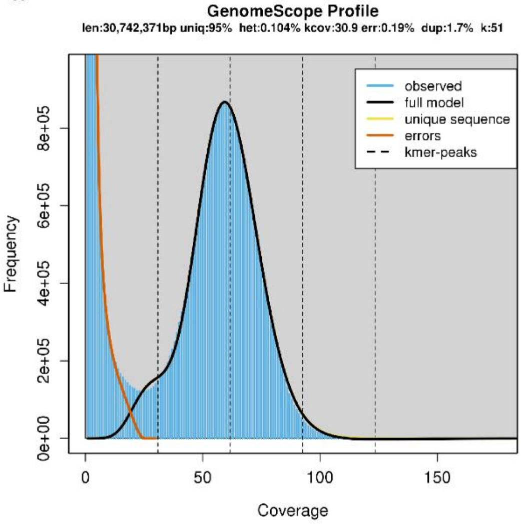


**b**

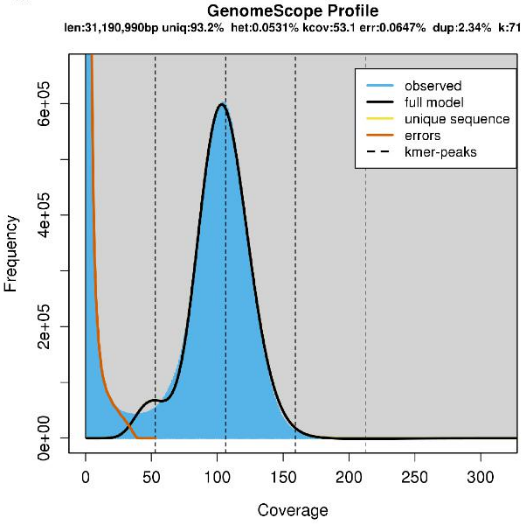

## Supplement 8. Genome assembly process in HN

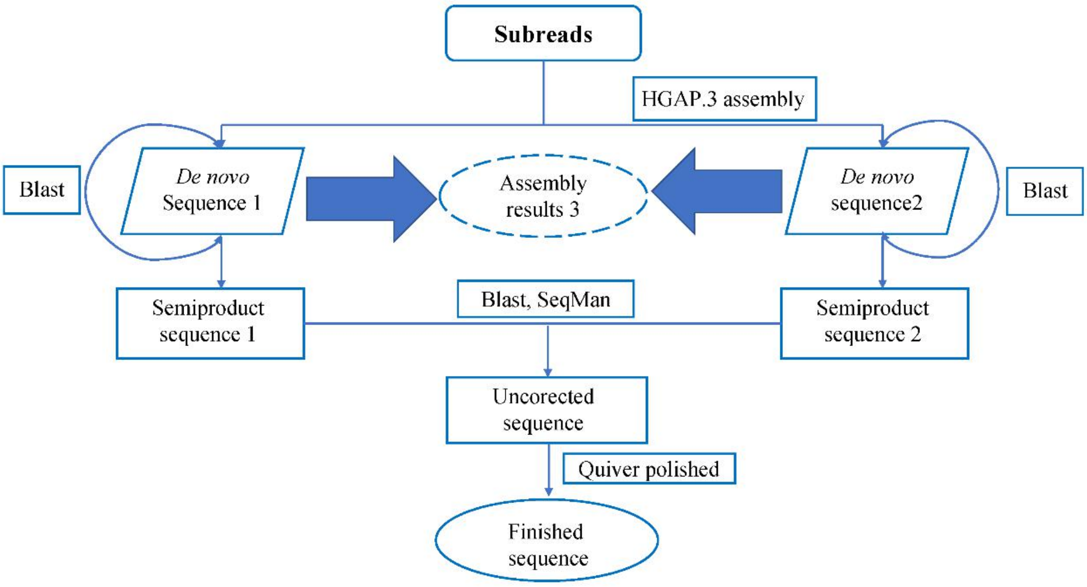

Supplement: Supplementary file 1 — Supplement information [file 41598_2018_38021_MOESM1_ESM.pdf]
